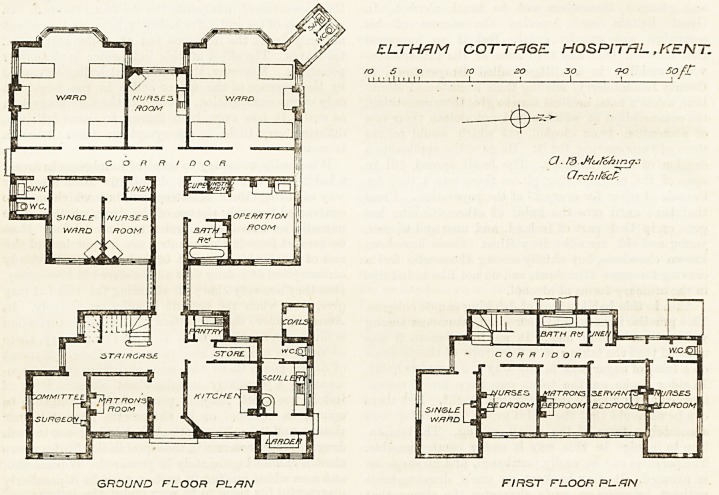# Hospital Construction

**Published:** 1900-01-20

**Authors:** 


### THE INSTITUTIONAL WORKSHOP.

### HOSPITAL CONSTRUCTION.

### ELTHAM COTTAGE HOSPITAL, KENT.

THE administration block faces east. Immediately on the left is the matron's sitting-room, and next to it is a room which serves the double purpose of committee room and surgeon's room. The kitchen offices are on the right hand; they include kitchen, larder, scullery, store, &c., and are arranged in the most compact and convenient manner. This department has a separate entrance to the north. The upper floor of this block contains a single-bedded ward, bedrooms for the staff, bath, and linen-room. A short, well-lighted corridor passes west, and connects the administration with the

GROUND FLOOR PLAN

FIRST FLOOR PLAN

hospital proper, and the extreme end of this corridor joins another which runs north and south, and divides the hospital block into two parts, thus affording an admirable medium for ventilation, as it has a door at the south end and a window at the north end. This corridor is one of the best features of the hospital. On its east side is the operation-room, with a large window to the north and a smaller one to the east; but there does not appear to be any roof-light—a serious omission. A bath-room adjoins the operation-room, and there is a good instrument cupboard in the room itself. We should like to have seen the sink removed to the party wall near the bath, the fireplace to the angle where the sink is, and an alternative exit from the operation-room to the main corridor thus obtained. Passing southwards along the main corridor we come to a linen room, a nurses' room, and a single-bedded room. On the west side of the corridor are the wards. These contain four beds each, and are well lighted; but it must be pointed out that the cross-ventilation is not so well provided for as it might have been by running the two wards eight feet further west, or by connecting the wards by subsidiary corridors placed at right angles to the main corridor. The north-west ward has its closet accommodation properly cut off by a cross-ventilated passage; but the south-west ward has its closet placed between the walls of the corridor and the single-bedded ward. This is a mistake. The closet, if placed near that position at all, ought to have been entirely separated from the angle by carrying the main corridor six feet further south. The building was erected in 1897, and opened in 1898 by Sir Henry Burdett. The hospital stands in half an acre of land. The elevations are in red brick, and the roof covered with tiles. The drainage is connected to the main sewer; and the water is provided by the Kent Waterworks. The architect is Mr. A. B. Hutchings, of Victoria Street, Westminster; and the contractors were Messrs. White and Sons, of Chislehurst. The building cost under £3,000.

## Figures and Tables

**Figure f1:**